# Assessing Medical Student’s Ability to Interpret Traumatic Injuries on Computed Tomography Before and After the Third Year Clerkships

**Published:** 2018-11-29

**Authors:** Brady Werth, Barbara Nguyen, Jeanette Ward, Jared Reyes, Stephen D. Helmer, Joseph Nold, Nicholas Brewer, James Haan

**Affiliations:** 1 University of Kansas School of Medicine-Wichita, Department of Surgery, Wichita, KS; 2 Chandler Regional Medical Center, Department of Trauma Services, Chandler, AZ Via Christi Hospital Saint Francis, Wichita, KS; 3 Department of Medical Education; 4 Department of Trauma Services; 5 Department of Radiology

**Keywords:** trauma, computed x-ray tomography, undergraduate medical education, radiolography

## Abstract

**Introduction:**

Exposure to radiologic images during clinical rotations may improve students’ skill levels. This study aimed to quantify the improvement in radiographic interpretation of life-threatening traumatic injuries gained during third year clinical clerkships (MS-3).

**Methods:**

We used a paired-sample prospective study design to compare students’ accuracy in reading computed tomography (CT) images at the beginning of their third year clerkships (Phase I) and again after completion of all of their third year clerkships (Phase II). Students were shown life-threatening injuries that included head, chest, abdomen, and pelvic injuries. Overall scores for Phase II were compared with Phase I, as well as sub-scores for each anatomical region: head, chest, abdomen, and pelvis.

**Results:**

Only scores from students participating in both Phase I and Phase II (N = 57) were used in the analysis. After completing their MS3 clerkship, students scored significantly better overall and in every anatomical region. Phase I and Phase II overall mean scores were 1.2 ± 1.1 vs. 4.6 ± 1.8 (p < 0.001). Students improved the most with respect to injuries of the head and chest and the area of least improvement was in interpreting CT scans of the abdomen. Although improvements in reading radiographic images were noted after the clerkship year, students accurately diagnosed only 46% of life-threatening images on CT scan in the trauma setting.

**Conclusions:**

These results indicated that enhanced education is needed for medical students to interpret CT scans.

## INTRODUCTION

No published evidence currently exists demonstrating improvement in a medical student’s ability to read and interpret traumatic radiographic images during their clinical rotations. Intuitively, exposure to radiologic images during clinical rotations should improve medical students’ skill with regard to reading and interpreting images of life-threatening injuries. However, as of 2009 - 2010, only a quarter of United States medical students were required to complete clinical rotations in radiology.^1^ In contrast to those statistics, the majority of undergraduate medical students surveyed by Saha et al.^2^ believed that becoming proficient in radiology was necessary to become a “competent doctor”. Additionally, a majority of General Surgery program directors believe it is essential for a surgeon to recognize common abnormal findings on abdominal x-ray, and to have a systematic approach of viewing a CT of the abdomen and pelvis.^3^,^4^

Exposure to radiology in the clinical or hospital setting, even when implemented in the early phases of medical education, can influence how students perceive an image and its subsequent interpretation.^3^,^5^,^6^ However, investigations have varied with regard to incorporating image reading and interpretation into curricula. Lufler et al.^6^ provided medical students with CT scans of their cadavers during their anatomy course. Students who had access to the CT scans were 3.6 times more likely to score greater than 90% on their exam than the students who did not have access. Another study incorporated a web-based radiology curriculum during students’ clinical clerkships.^3^ Surveys from the students showed that 88% of the students found this course expanded their knowledge and understanding of radiology; however, no objective data were examined with this study, or other studies, to show evidence of improvement.

Residents believe that a working knowledge of radiology is important. Saha et al.^2^ conducted a survey of interns and found that two-thirds are asked to make a preliminary diagnosis on multiple modes of imaging several times a week. Of the residents surveyed, 93.4% thought it important to be able to read a chest x-ray accurately as an intern, and 79.3% thought it important to interpret abdominal radiographs correctly. Program directors in General Surgery list the reading and interpreting of radiological images as a skill that is important, but 41.7% indicated that incoming residents lack adequate radiology skills and knowledge.^4^ This means that interns will be expected to interpret images, sometimes on their own, whether program directors think they have adequate knowledge or not.

No formal objective study has been performed to ascertain level of improvement made in identifying life-threatening traumatic injuries on CT scan. Therefore, the purpose of this study was to observe and compare the baseline knowledge of CT interpretation of traumatic injuries for medical students before and after completing their clinical clerkships. Objectively evaluating a cohort of students before and after their required clinical clerkships will guide future discussions of improving the radiologic knowledge of medical students, especially during their clerkships as they prepare to enter residency.

## METHODS

### Study setting

This study took place at the University of Kansas School of Medicine-Wichita, which utilizes a four-year program of study. The first two years are didactic and the second two are composed of clinical clerkships and clinical electives. During the third year surgery clerkship, students are exposed to trauma call on the weekends and periodically on overnight trauma call.

Students are integrated into all aspects of the trauma experience, and trauma residents are instructed to teach students how to interpret CT imaging in the trauma situation. A majority of students received a lecture by a trauma surgeon during their third year specifically designed to teach them how to interpret CT scans in the trauma setting. Many times in overnight trauma call, trauma residents are expected to make a preliminary diagnosis based on imaging, which guides clinical decision-making.

### Study selection and consent procedures

The Institutional Review Board of the sponsoring hospital approved this study for implementation. Study participants were medical students of the University of Kansas School of Medicine-Wichita who voluntarily consented to participate. A cohort of third-year medical students were asked to participate in a timed, open-ended survey just prior to beginning their third year clerkships (MS-3) to assess their ability to diagnose life-threatening injuries on CT scan that were typical of the trauma population. The survey was conducted during a scheduled time of their orientation for clinical clerkships, but was not a mandatory part of their curriculum. Students of the same cohort again were asked to participate voluntarily in a similar survey after completion of all of their third year clerkships in transition between their third and fourth year of medical school (MS-4). Informed consent was obtained from all volunteers for both surveys, and they were informed that they were under no obligation to participate and that all answer sheets would be de-identified.

### Radiographic image selection and survey procedure

The trauma registry of an American College of Surgeons-verified Level 1 trauma center was used to find representative images of different traumatic injuries diagnosed by CT. The final images to be used in the study (Tables 1 and 2) were selected by a fellowship-trained trauma surgeon with 11 images being selected for MS-3 (Phase I) and 10 for MS-4 (Phase II) students. Images chosen were representative of four different anatomical regions: head, chest, abdomen, and pelvis. The images chosen were deemed to represent the injuries to be identified and as such should have minimized the need to scroll through the images as would be available in real life. The images were de-identified and loaded onto Microsoft^®^ PowerPoint slides for viewing. There were an unequal number of CT images used in each phase (11 in the MS-3 session; 10 in the MS-4 session). Since a comparable colon mesenteric injury was not presented after completion of the third year clerkship, scored answers for that image were removed from analysis. The students were gathered in a theater style lecture hall, instructed that each slide represented a life-threatening injury, and given two minutes to render their answers on each injury. The students were not allowed to discuss the images with each other; it was a test of their independent ability. They were asked two questions on each injury for a total of 22 questions for MS-3 and 20 for MS-4 students: what was the diagnosis and what intervention would they recommend for the injury.

### Survey scoring and data analysis

After completion of the survey, the forms were de-identified and scored independently by two trauma surgeons. The surgeons then reviewed the scores and disagreement in scoring was reviewed and resolved by mutual agreement and a final score for each student determined. Students were given one point for a correct response, one-half point for a partially correct response, and zero points for incorrect answers. Students were scored only on the first question (What was the diagnosis?).

The second question (What intervention would they recommend for the injury?) was not scored, as the correct answer to this question is dependent on first getting the correct diagnosis. The mean score with standard deviation was calculated for the four anatomical regions that were tested in both sessions, as well as the overall score in each of the two sessions. For analysis, comparisons of continuous data were conducted using a Wilcoxon signed rank test due to the skewed nature of the assessment scores. All statistical tests were two-sided and analyses were considered significant when the resultant p value was ≤ 0.05. All analyses were conducted using SPSS release 19.0 (IBM Corp., Armonk, New York).

## RESULTS

A total of 65 MS-3 students agreed to participate in the study. Of those, 57 students were re-surveyed with a similar survey as MS-4 students. Overall, the cohort scored considerably better as MS-4s than as MS-3s (Table 3). The overall MS-4 score was 4.6 compared to 1.2 as MS-3s out of 10 possible. Statistically significant improvement was made by the cohort in every anatomical region tested with all p values less than or equal to 0.005 (Table 3). The largest improvement was made in interpreting CT images of the chest (0.14 vs. 1.45, p < 0.001). The smallest improvement was made in interpreting CT images of the pelvis (0.25 vs. 0.52, p = 0.005). Specific injuries tested in each anatomical region are in Tables 1 and 2.

## DISCUSSION

Integrating the appropriate radiological training into medical school has long been a source of debate. This debate may be expected to continue as the use of diagnostic imaging has been increasing dramatically over the previous few decades.^18^ There have been studies to show that medical students feel that they increase their ability to diagnose common abnormalities with training.^1^–^3^ Interns of multiple specialties often are asked to render preliminary diagnoses and identify common pathologies such as pneumonia, small bowel obstruction, pneumoperitoneum, and intracranial hematoma.^2^ The ability to diagnose gross life-threatening injuries quickly is important on a trauma service where many injuries happen at night, access to radiologists is limited, and clinical decisions largely based on imaging must be made to direct patient care decisions.

Our study showed that students’ ability to identify life-threatening images on CT scan improve during their third year clerkships. However, they still misidentify a substantial number of injuries. Students performed best when evaluating head injuries, while the area in which we observed the greatest improvement was that of chest CTs. Students’ largest deficit of knowledge appeared to be in identifying abdominal injuries. This likely was reflective of the complexity of the abdomen with multiple organ systems; however, this finding also highlighted the need for continued radiological exposure and training.

The trauma service that this cohort was exposed to only allowed for specific trauma training in regards to CT interpretation during the students’ on call duties, yet they still showed improvement. It was also evident that this same cohort of students had a strong deficit of knowledge when interpreting CT and required more training. This training could come from multiple sources, but an organized approach to teaching and evaluating students is critical to ensuring all students are receiving similar experiences. Students at this institution have the option of an elective radiology rotation during their MS-4 year, which could improve this skillset. The majority of this cohort was subjected to a dedicated lecture on interpreting CT scans in trauma. A web-based radiology curriculum, such as was instituted by Chorney and Lewis^3^ seemingly could be useful and has the perception of being a good use of resources and time, and provides increase in knowledge by the subjects included in the studies’ surveys. Studies comparing students’ ability to interpret radiological pathology after implementing a web-based curriculum should be undertaken.

Several limitations are evident in this study. This study was performed in one small cohort at a single institution, thus its generalizability to non-similar programs and students may be lessened. Images representative of injuries on CT were presented to the students as a single slide to the entire cohort simultaneously. This is not a scenario representative of clinical practice. Having the ability to scroll through images and look at different reconfigurations clearly is helpful in identifying abnormal anatomy and the magnitude of injury. If students had the ability to scroll through the CT scans as they would in clinical practice their identification of injuries may have been higher. However, the images shown were chosen for their clarity and the need to mitigate this limitation. Students rotate through the surgery service at different times of the year, with different residents teaching them. This adds variability to each set of students experience in trauma. The curriculum is built to be the same, but we could not control for quality of resident teaching, nor of seasonal injuries. Most students only exposure to trauma was during their on-call duties, but one to two students in each rotation were placed on the trauma service, and spent a dedicated month on trauma, greatly increasing their exposure. This was not taken into account in this study. Finally, there is a possibility of selection bias resulting from performance differences in students who chose to volunteer vs those who did not; however, we believe this is unlikely to have affected our results. With an average of approximately 70 students in each class, the vast majority of students participated in our study, making it unlikely that any effect on our results would significantly affect our interpretations and conclusions.

## CONCLUSIONS

Standard methods for evaluating medical students’ ability to interpret radiographic imaging are lacking. In addition, the best practices to facilitate proficiency in evaluating radiographic imaging still are debated. This study showed that medical students improve in their ability to identify life-threatening traumatic injuries on CT during the course of clinical rotations. However, improved though they may be, a deficit remains between acquired skill and what may be expected as interns.

In one year, those students will be expected to render preliminary diagnoses based on CT and implement clinical interventions based on their interpretations as intern residents. This study suggested that medical students need focused training in interpreting CT scans. The method of this training and standards used to evaluate the student should be the subject of future study.

## Figures and Tables

**Table 1:** Life-threatening computed tomography injuries used for survey 1 for MS-3 students.

Anatomical Area	Injury
Head	Epidural Hematoma Subdral Hematoma Intraparenchymal Hemorrhage
Chest	Pulmonary Contusion and Pneumothorax Pulmonary Contusion and Pneumothorax
Abdomen	Liver Laceration Splenic Laceration Renal Injury Small Bowel Thickening Colon Mesenteric Injury*
Pelvis	Pelvic Fracture

* This image was not used in the paired comparison for abdominal injuries, as a comparable injury image was not shown during the MS-4 survey.

**Table 2:** Life-threatening computed tomography injuries used for survey 2 for MS-4 students.

Anatomical Area	Injury
Head	Epidural Hematoma Subdral Hematoma Intraparenchymal Hemorrhage
Chest	Pulmonary Contusion and Pneumothorax Pulmonary Contusion and Pneumothorax
Abdomen	Liver Laceration Splenic Laceration Renal Injury Small Bowel Thickening and Renal Injury
Pelvis	Pelvic Fracture

**Table 3:** A comparison of correct CT interpretation scores for medical students in years 3 and 4 (n = 57).

Body Region	Maximum Possible Score	Mean Score ± SD		Significance^a^
		MS3	MS4	
Overall	10	1.16 ± 1.00	4.62 ±1.75	p < 0.001
Head	3	0.74 ± 0.56	1.83 ± 0.83	p < 0.001
Chest	2	0.14 ± 0.44	1.45 ± 0.66	p < 0.001
Abdomen	4	0.11 ± 0.34	0.83 ± 0.91	p < 0.001
Pelvis	1	0.25 ± 0.43	0.52 ± 0.49	p = 0.005

a Non-parametric test of significant differences were used due to the atypical distribution of one or more of the comparison groups.
